# Evaluation of serum procathepsin B, cystatin B and cystatin C as possible biomarkers of ovarian cancer

**DOI:** 10.3402/ijch.v72i0.21215

**Published:** 2013-08-05

**Authors:** Elena A. Gashenko, Valentina A. Lebedeva, Ivan V. Brak, Elena A. Tsykalenko, Galina V. Vinokurova, Tatyana A. Korolenko

**Affiliations:** 1 Institute of Physiology SB RAMS, Novosibirsk, Russia; 2 Municipal Hospital N 3, Novosibirsk, Russia; 3 Department of Oncology, Novosibirsk Medical University, Novosibirsk, Russia; 4 Regional Diagnostic Center, Novosibirsk, Russia

**Keywords:** ovarian cancer, procathepsin B, cystatin B, cystatin C, biomarkers

## Abstract

**Objectives:**

To evaluate procathepsin B, as well as endogenous inhibitors of cysteine proteases (cystatin B and cystatin C) in biological fluids as possible biomarkers of ovarian cancer. To observe levels of serum procathepsin B in different age groups.

**Study design:**

The sample (N=27) of women with gynaecological tumours included 18 patients with ovarian cancer (n=18) and 9 patients with benign ovarian tumours (n=9); 72 healthy women were in the control group. All patients were treated in Novosibirsk Regional Oncological Center, Russia. Serum samples of healthy women (n=40) aged 18–70 years were used as controls for common biomarker of ovarian cancer CA-125. In the Procathepsin B study, serum samples of healthy women (n=32) aged 18–40 years (n=14), 41–55 years (n=10) and 56–80 (n=8) years were used as controls.

**Methods:**

Common biomarker of ovarian cancer, CA-125, was assayed by using a commercial kit (Vector, Koltsovo, Novosibirsk Region, Russia). Procathepsin B was measured by means of a commercial kit for human procathepsin B (R&D, USA); cystatin C was measured by commercial ELISA kits for human (BioVendor, Czechia); cystatin B was measured by ELISA kits for human (USCN Life Science Inc., Wuhan, China). Statistical analysis was performed by one-way ANOVA (Statistica 10 Program).

**Results:**

In the control group, serum procathepsin B concentration did not reveal age dependency. In the ovarian cancer group, both levels of serum procathepsin B and standard biomarker CA-125 increased significantly (both p<0.001) compared with the control group. In the benign ovarian tumour group, serum procathepsin B (p<0.001) and CA-125 (p=0.004) increased about 2.5- and 8-fold compared to the control group. Serum cystatin B level increased up to 1.7-fold in the ovarian cancer group compared to the control group. The increase of serum CA-125 was about 3.5-fold higher (p=0.017) and procathepsin B was 1.8-fold higher (p<0.05) in the ovarian cancer group compared to the benign tumour group. Cystatin B in ascites fluid increased equally in both ovarian cancer (p<0.001) and benign ovarian tumours group (p<0.05). Cystatin C concentration in *ascites* fluid increased only in patients with ovarian cancer (p<0.05) and did not change in the benign tumours group. Large increases of procathepsin B level (about 13-fold, p<0.001) and to a lesser degree of cystatin C (1.8-fold, p<0.05) and cystatin B levels (1.4 fold, p<0.001) were revealed in ascites fluids of patients with ovarian cancer compared to the control serum.

The significant difference in serum procathepsin B levels was noted between the ovarian cancer and benign tumour groups (p<0.05), which could be used in differential diagnostics between malignant and benign gynaecological tumours.

**Conclusion:**

Serum procathepsin B demonstrated significant promise as a new biomarker of ovarian cancer.

Ovarian carcinogenesis remains a research challenge due to many unknown factors involved in the malignant transformation sequences (1,2). Tumour cells were able to secrete both mature and proforms of proteases, and their inhibitors (to protect themselves from proteolysis) (3,4). Proteases of different classes (cysteine, serine, aspartate and matrix metalloproteases) were universally involved in tumour development (5). Cysteine proteases (cathepsins B, L, H, K, S) and their endogenous inhibitors, cystatins, had been shown to play an important role both in tumour growth and metastases (6,7).

CA-125, cancer antigen 125, was known as a protein that was found at high levels in most ovarian cancer cells. However, CA-125 was also elevated in several benign tumours.

Procathepsin B was a pro-form of active mature form of cathepsin B (8,9) and could be suggested as a new tumour biomarker in ovarian cancer.

Cystatin C and cystatin B, endogenous inhibitors of cysteine proteases, possibly were involved in tumour growth and cell proliferation (6,10). Cystatin B was thought to play a role in protecting against the cysteine proteases “leaking” from lysosomes (11) and was related to the prognosis of colorectal cancer (12,13), while cystatin C was involved in the regulation of cell proliferation, differentiation and migration (14). However, until now the role of cystatins B and C in human pathology, especially in oncology, had not been well understood. Searching for new tumour biomarkers among mature forms of proteases, their enzymatically inactive proforms and endogenous inhibitors of proteases is an important step in cancer diagnostics.

## Materials and methods

### Study design

All participants were divided into the following groups: ovarian cancer (n=18), benign tumours (n=9), which included serous cystadenoma, mucinous cystadenoma and dermoid cyst) and a control group (n=72). All patients were treated at Novosibirsk Regional Oncological Center (Department of Oncological Gynecology and Department of Mammology). Informed consent of patients was obtained in all cases; the Ethical Committee of the Institute of Physiology of SB RAMS approved the research. In procathepsin B study, serum samples of healthy women (n=32) aged 18–40 years (n=14), 41–55 years (n=10) and 56–80 (n=8) years were used as controls. Serum samples of healthy women (n=40) aged 18–70 years were used as controls for common biomarkers of ovarian cancer CA-125.

*Serum and ascetic fluids* of women with tumours of the reproductive system (aged 18–80 years) before operation were used for assay of procathepsin B, cysteine protease inhibitors and CA-125. Serum was obtained after centrifugation of blood samples at 3,000×*g* for 20 min at 4°C (Eppendorf centrifuge 5415R, Hamburg, Germany) and stored at −70°C until analysis. Ascetic fluid was obtained by syringe before surgery.

CA-125 (CA-125-Immynoassay-Best kit, Vector, Koltzovo, Novosibirsk Region, Russia) was measured by Bio-Rad photometer, Model 68.

Procathepsin B concentration in serum and ascetic fluid was measured by ELISA kits (R&D) for quantitative assay of human procathepsin B; cystatin B concentration was measured using ELISA kits for quantitative assay of human cystatin B level (USCN, Life Science Inc., China); and finally, cystatin C concentration was measured using ELISA kits for quantitative assay of human cystatin C (BioVendor, Czech Republic).

*Human procathepsin B* immunoassay, a solid phase of ELISA, was designed to measure the pro-form of cathepsin B in different biological fluids. The minimum detectable dose of pro-cathepsin B ranged from 0.003 to 0.079 ng/ml.

*Human Cystatin B*. ELISA kit for assay of human cystatin B (with detection range 31.2–2,000 pg/ml and sensitivity 12.6 pg/ml was used.

*Human Cystatin C*. ELISA kit for human cystatin C with detection range 0.312–1,000 ng/ml and sensitivity 10 pg/ml was used.

Statistical analysis was performed by one-way ANOVA using Statistica 10 Program and Student t-test; the data were presented as mean±SEM; the difference was significant at p<0.05.

## Results

**Fig. 1:**
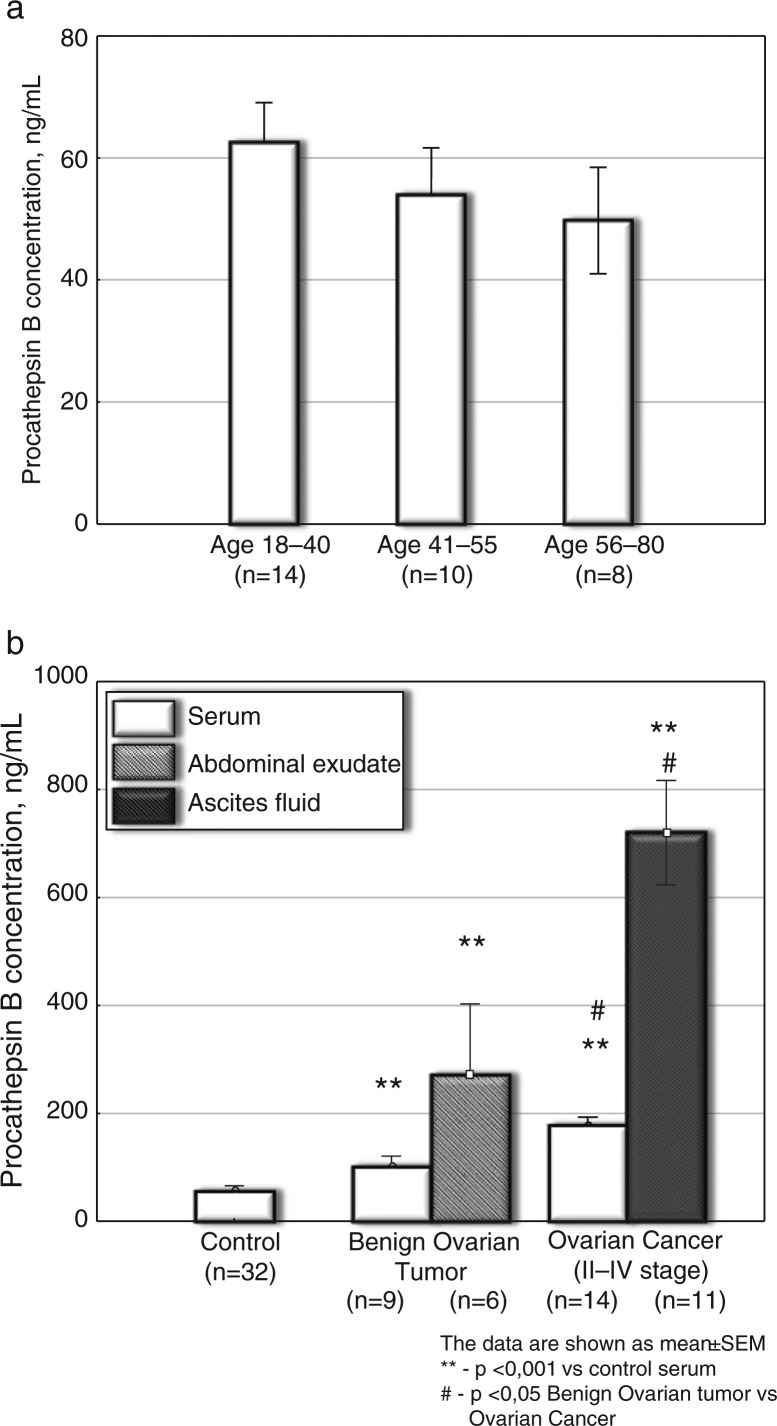
Procathepsin B concentration in serum and ascetic fluids of patients with ovarian cancer and benign tumours. a. Procathepsin B in serum of healthy persons of different age (ng/ml). b. Procathepsin B in serum and ascetic fluids of patients with ovarian cancer and benign tumours (ng/ml). The data are shown as mean±SEM. The number of patients is in parentheses.

**Fig. 2:**
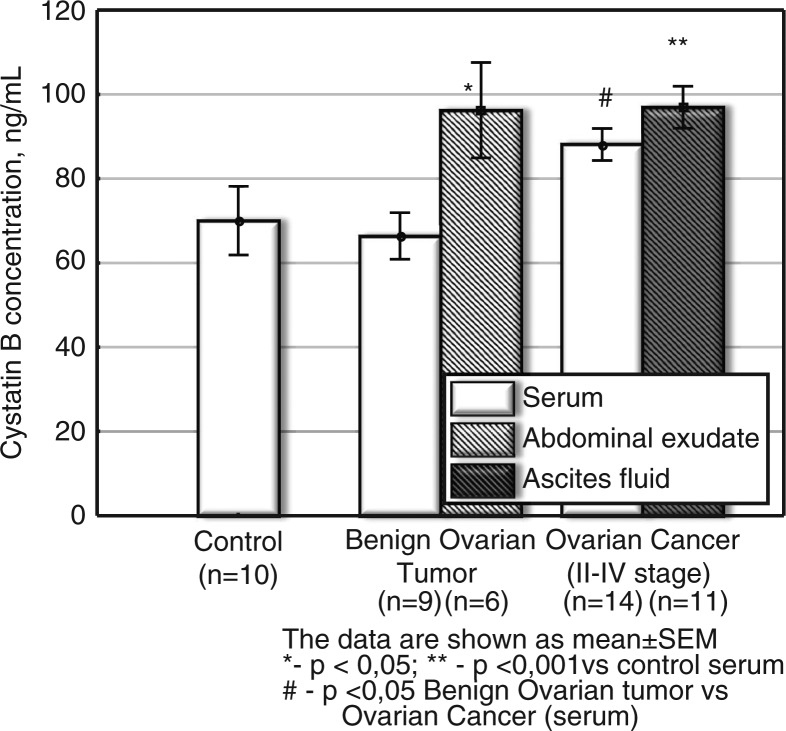
Cystatin B concentration in serum and ascetic fluids of patients with ovarian cancer and benign tumours (ng/ml). The data are shown as mean±SEM. The number of patients is in parentheses.

**Fig. 3:**
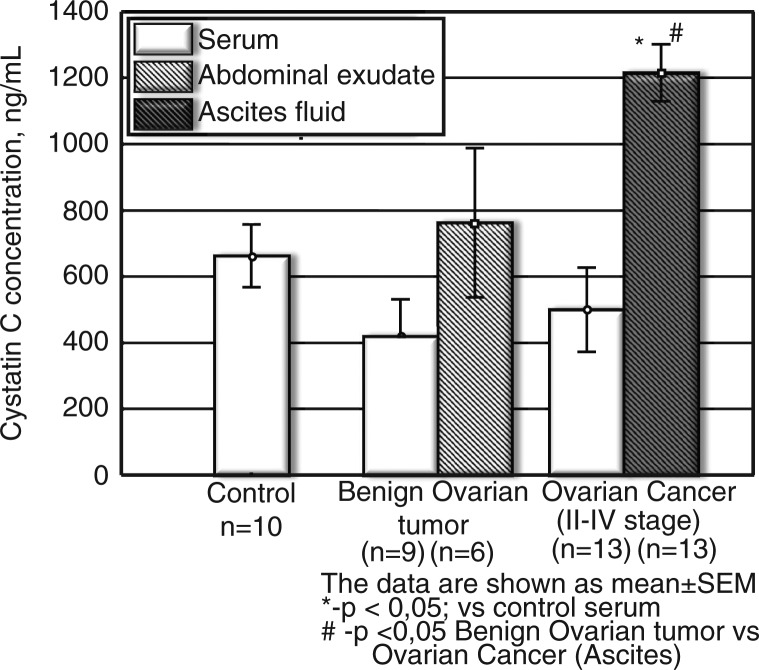
Cystatin C concentration in serum and ascetic fluids of patients with ovarian cancer and benign tumours (ng/ml). The data are shown as mean±SEM. The number of patients is in parentheses.

The results showed the following: the increase of procathepsin B concentration in ascites of patients with ovarian cancer was the highest compared to the serum of controls (elevation about 13-fold, p<0.001) (Fig. 1); with cystatin C being the second highest (p<0.05, elevation about 1.8-fold) (Fig. 3) and cystatin B being the third highest (p<0.001, increase up to 1.4-fold) (Fig. 2).

We have shown that in the serum of healthy persons, CA-125 concentration was below 30–35 ng/ml (8.4±1.3 ng/ml), whereas in the ovarian cancer group serum, CA-125 level increased significantly (276.9±45.7 ng/ml, p<0.001) as was observed earlier by other authors (1,15). However, CA-125 concentration in ovarian cancer was only about three times higher compared to benign ovarian tumours (77.9±52.6, n=9, p<0.05).

### 

#### Procathepsin B

In the control group of practically healthy persons aged 18–40 years (14 persons), 41–55 years (10 persons) and 56–80 years (8 persons), serum *procathepsin B* concentration did not reveal age dependency (Fig. 1a). A tendency towards a decreased level of serum procathepsin B was observed in the group of elder patients (56–80 years) (Fig. 1a). The significantly increased concentration of procathepsain B up to 186.4±23.48 ng/ml was revealed in the serum of patients with ovarian cancer, p<0.001 (Fig. 1b), especially in the cases of ascites-producing ovarian tumours. In the ovarian cancer group, both levels of serum procathepsin B and CA-125 significantly increased (both p<0.001) compared to the controls. Significant (p<0.001) elevation in procathepsin B was also noted in ascetic fluids of patients with ovarian cancer −660.3±97.20 ng/ml, n=11 (Fig. 1b). In the ovarian cancer group, the increase in serum procathepsin B concentration versus the control serum (p<0.001) was similar to the increase in CA-125 level versus the controls (p<0.001).

In the benign ovarian tumour group, elevated serum procathepsin B (p<0.001) and CA-125 (p=0.004) had similar values as tumour biomarkers versus serum of the control group. The increase of serum CA-125 concentration was greater compared to the increase of procathepsin B level in the ovarian cancer group.

Milder elevation of serum (p<0.001) and ascetic (p<0.001) procathepsin B concentration (versus the control serum) was shown in patients with benign gynaecological tumours (Fig. 1b). The significant difference in serum procathepsin B levels was noted between the ovarian cancer and the benign tumour groups (p<0.05, Fig. 1b), which can be used in differential diagnostics between malignant and benign gynaecological tumours. One can conclude that assay of procathepsin B in serum is a promising method of ovarian cancer diagnosis and differentiation between the malignant and benign ovarian tumours.

#### Cystatin B

In general, the change in *serum* protease inhibitor *cystatin B* was milder (Fig. 2) as compared to CA-125 and procathepsin B. Moreover, serum cystatin B level had a tendency to increase (p<0.05 vs. serum in the control group) only in ovarian cancer, not in benign ovarian tumours (Fig. 2). Earlier, according to the literature, cysteine protease inhibitors (cystatins C, A and B) were found in ascetic fluids of patients with ovarian carcinoma (7). It was suggested that the inhibitors had possible diagnostic/prognostic value. In our study, the level of cystatin B increased in abdominal exudate in benign tumours (compared to the control serum, p<0.05), as well as in ascetic fluids (p<0.001 vs. serum of the control group) in ovarian cancer of stages II–IV (Fig. 2). There was no significant change in *serum* cystatin B in the benign tumour group versus control serum. Simultaneously, serum concentration of cystatin B in ovarian cancer (stages II–IV) was slightly elevated (p<0.05) versus serum of patients with a benign tumour (Fig. 2).

#### Cystatin C

*Serum* cystatin C concentrations in the ovarian cancer and benign tumour groups did not differ significantly (Fig. 3). However, in ascetic fluids cystatin C increased versus control serum level (p<0.05), as well as benign ovarian tumours (p<0.05). A significant difference (p<0.05, Fig. 3) between cystatin C level in ascetic fluids of persons with ovarian cancer and a benign tumour can be potentially useful in differential diagnosis of those diseases.

## Discussion

According to the literature, cystatin C, as well as mature cathepsin B, were involved in the mechanism of invasion of ovarian cancer (16,17); however, this mechanism was not well understood.

It was suggested that the *serum* cystatin C changes in ovarian cancer were minor and unrelated to the diagnosis or prognosis of this tumour. At the same time, one can argue that in the diagnosis or prognosis of ovarian cancer, the study of cystatin C in *ascetic fluids* might be more important than in serum. Further research is necessary in this field.

Earlier it was shown that in patients with ovarian cancer, the changes of cysteine protease inhibitor cystatin C were not completely associated with cysteine protease cathepsin B. About 20% of high-molecular-weight inhibitors and 50% of low-molecular-weight inhibitors were free in native ascites fluid (7). Recently, cystatin C was suggested as an independent prognostic factor for the survival in some tumours (18). The authors had shown that serum cystatin C was not only a sensitive marker of renal impairment, but also related to tumour burden and prognostic value in myeloma. Microarray analysis had revealed that cystatin C was one of the most highly upregulated genes in multiple myeloma. Its reduction after treatment with bortezomib reflects bortezomib's anti-tumour activity (18).

According to our results, cystatin C increased only in ascetic fluid (not in serum) of patients with ovarian cancer and did not increase in the benign tumour group. This fact requires further investigation. It was shown in the presented work that compared to serum of the control group, the increase in procathepsin B concentration (p<0.001,13-fold elevation) in ascites of patients with ovarian cancer was the highest; with cystatin C being the second highest (p<0.05, elevation about 1.8-fold) and cystatin B the third highest (p<0.001, increased up to 1.4-fold).

During tumour invasion and metastasis, limited degradation of extracellular matrix was mediated by different proteases, especially by lysosomal cysteine protease cathepsin B, which can activate other proteolytic enzymes, such as urokinase-type plasminogen activator and collagenase I (19–22). Tumour cells secreted procathepsin B and both active forms of cathepsin B and cysteine protease inhibitors (23–26). Secretion of procathepsin B occurred as a result of increased expression, whereas secretion of active cathepsin B seems to involve active processes that can be induced by a variety of mechanisms. Once secreted, procathepsin B binded to the tumour cell surface via p11, the light chain of the annexin II heterotetramer (19,20). This binding of procathepsin B seems to facilitate tumour invasion and metastasis (19).

## Conclusion

Serum and ascetic procathepsin B had demonstrated significant promise as a new biomarker of ovarian cancer. The obtained data supported the hypothesis about the important role of enzyme pro-forms and endogenous inhibitors of cysteine proteases in tumour growth.
